# Tryptophan-driven metabolomic shift in *Acidobacteriaceae* reveals phytohormones and antifungal metabolites

**DOI:** 10.1128/msystems.00828-26

**Published:** 2026-09-14

**Authors:** Celine M. Zumkeller, Christoph Hartwig, Walter Lanzalonga, Maria A. Patras, Yang Liu, Michael Marner, Sanja Mihajlovic, Marius S. Spohn, Till F. Schäberle

**Affiliations:** 1 Institute for Insect Biotechnology with Focus on Natural Product Research, Justus-Liebig-University Giessen, Giessen, Germany; 2 Natural Product Department, Fraunhofer Institute for Molecular Biology and Applied Ecology (IME-BR) 318347, Giessen, Germany; 3 German Center for Infection Research (DZIF), Partner Site Giessen-Marburg-Langen, Giessen, Germany; Wageningen University, Wageningen, the Netherlands

**Keywords:** *Acidobacteriota*, phytohormones, *N*^6^-(Δ^2^-isopentenyl)adenine, iP, indole-3-acetic acid, IAA, PGPT, BGC

## Abstract

*Acidobacteriota* is one of the most abundant phyla in soils and has recently attracted attention for its potential role in promoting phytosanitary benefits. The metabolomic capabilities of this phylum remain poorly characterized, with few experimentally confirmed metabolites described. To address these gaps, we combined untargeted metabolomic profiling with comparative genomic analyses to explore the functional potential of newly isolated *Acidobacteriaceae* strains. Genome mining across the *Acidobacteriota* phylum revealed the presence and taxon-specific enrichment of genes associated with plant-related traits, including phytohormone biosynthesis. In parallel, metabolomic analyses of OSMAC-derived extracts uncovered pronounced condition-dependent metabolic variation. Tryptophan supplementation was associated with marked metabolomic reprogramming, including changes in indole-derived metabolites, such as indole-3-acetic acid. Subsequent analyses linked these metabolic shifts to the suppression of phytopathogenic fungi and enabled the identification of malassezindoles and pityriacitrins as active compounds, confirmed by structure elucidation using NMR spectroscopy. Overall, these findings shed light on the previously unexplored metabolic potential of the *Acidobacteriota* phylum, emphasizing its ecological importance for phytosanitary applications.

**IMPORTANCE:**

Despite their ubiquity and genomic diversity, the functional metabolism of members of the *Acidobacteriota* has largely remained uncharacterized. This study links genomic predictions to experimentally verified metabolomic outputs of *Acidobacteriaceae*, demonstrating tryptophan-responsive metabolic shifts translating to phytohormones and metabolites suppressing fungal growth. Our work underscores the emerging role of *Acidobacteriota* as important contributors to soil ecosystem functioning and plant–microbe interactions.

## INTRODUCTION

Ubiquitous across various ecosystems, including soil (1–3), marine sediments (4), and wetlands (5–7), as well as extreme environments such as permafrost (8) and acidic mine drainages (9), *Acidobacteriota* represent one of the most widespread bacterial groups (10). The phylum is phylogenetically diverse (11) and is classified into 26 subdivisions based on 16S rDNA sequencing (12). Despite their ubiquity, the *Acidobacteriota* have historically been underexplored due to the limited availability of cultured representatives, which has restricted experimental characterization and genomic insights (13). Today, the availability of high-quality metagenome-assembled genomes, combined with the growing number of cultured representatives and isolate genomes, has significantly broadened our understanding of the metabolic capabilities and ecological roles of this phylum (13–15).

Initially described as slow-growing oligotrophs with limited metabolic dynamics (16), adapted to nutrient-depleted environments, modern insights now reveal their versatility and critical involvement in biogeochemical processes, including sulfur (17) and nitrogen (18) cycling, as well as H_2_ consumption (19). Furthermore, they can degrade plant-derived polysaccharides using their rich repertoire of carbohydrate-active enzymes, such as cellulases, chitinases, and xylanases (20). Finally, early studies dating back to 2006 proposed a substantial potential for secondary metabolite production by biosynthetic gene clusters, identifying novel polyketide synthase gene clusters of acidobacterial origin from soil (21, 22). Subsequent genomic (23) and metagenomic studies confirmed this finding, identifying biosynthetic-gene-cluster (BGC)-enriched lineages such as the *Acanthopleuribacteraceae* (24) and the genus *Candidatus Angelobacter* (22). Yet, translating bioinformatic findings into experimental validation remains difficult and still requires the availability of culturable representatives. Notably, Leopold-Messer et al. (24) successfully retrieved marine sponge symbiont strains from the *Acanthopleuribacteraceae* family, marking the first description of non-ribosomal peptide (NRP)-polyketide (PK)-derived secondary metabolites from this phylum (24).

To overcome these gaps, various cultivation approaches have been developed to selectively favor acidobacterial growth and retrieve single-isolate strains from complex environmental samples (3, 25–27). These approaches have significantly increased the number of isolated strains; however, they still only represent a fraction of the predicted phylum diversity. Most of the strain isolates are retrieved from soil (23), where *Acidobacteriota* have consistently shown high abundances, ranging from 20% to 40% of the detected 16S rRNA (28, 29) sequences. Cultivation strategies often utilize plant polymers for *Acidobacteriota* enrichment (25, 26, 30), providing insight into their predicted function in enhancing soil health by degrading undigestible plant polymers and improving nutrient content in the soil. Consequently, several studies investigate the potential plant growth-promoting (PGP) effects of the phylum. In 2016, the first evidence of a direct plant-interaction was described by Kielak et al. (31). They found that some acidobacterial strains produced IAA and also demonstrated a PGP effect on the model plant *Arabidopsis thaliana* (31). Moreover, *Acidobacteriota* are recognized for synthesizing hopanoids (32), bacterial analogs of sterols, known to enhance stress tolerance in soil bacteria as an integral component of the bacterial membrane. Their unique exopolysaccharide (EPS) production (33, 34) further suggests a potential ability to endure dry conditions and possible roles in sustaining plant health, e.g., protecting against desiccation. Complementary metagenomic and meta-transcriptomic studies have revealed a significant enrichment of genes related to PGP. Gonçalves et al. profiled 758 soil-associated metagenome-assembled genomes (MAGs) and identified plant growth-promoting traits (PGPTs) involved in phytohormone production, nitrogen fixation, phosphorus solubilization, siderophore production, and EPS biosynthesis (20). These traits were explicitly enriched in certain families of the *Acidobacteriota*, namely *Acidobacteriaceae*, *Pyrinomonadaceae*, and *Koribacteriaceae*. Meta-transcriptomic evidence shows that these traits are actively transcribed in their respective environmental context (20). Furthermore, transcriptomic research has demonstrated the metabolic dynamics and responsiveness to external stimuli of cultured *Acidobacteriota* strains. For instance, *Acidobacterium capsulatum* responds to oxygen deprivation by switching to a respiro-fermentative state, resulting in the production of acetate and ethanol—a strategy that allows for survival in habitats with fluctuating O_2_ levels, such as soil (35). The oligotrophic lifestyle typical of the *Acidobacteriota* has been investigated in a *Granulicella* species, which responds to high-carbon availability by upregulating stress-related genes, indicating sensitivity to nutritional changes (36). While many ecological functions are inferred from meta(transcriptomics) data, experimentally generated metabolomic data remain limited, primarily focusing on the characterization of membrane lipids and their production in response to different cultivation parameters such as temperature, pH, and O2 (37, 38).

To address these gaps, we integrated metabolomic profiling with comparative genomic analysis to investigate the functional potential of novel strains of the *Acidobacteriaceae* family. Phylum-wide genome mining for PGPTs revealed the presence and taxon-specific enrichment of genes associated with phytohormone production, as well as additional genes related to PGP effects. The addition of tryptophan (Trp) to the cultivation medium was associated with marked changes in metabolite profiles, resulting in quantitatively determined shifts in the production levels of the phytohormones indole-3-acetic acid (IAA) and N⁶-(Δ²-isopentenyl) adenine (iP). Prompted by these findings, we evaluated the metabolic reprogramming induced by Trp supplementation, revealing suppressive effects on the growth of phytopathogenic fungi and subsequently identified pityriacitrins and malassezindoles as the causative agents. Collectively, these findings highlight the previously underexplored metabolic space of the *Acidobacteriota* phylum, underscoring its ecological significance for phytosanitary purposes.

## RESULTS AND DISCUSSION

### Result 1: cultivation of *Acidobacteriaceae* shows condition-dependent extract diversity but no detectable antimicrobial activity

Extensive metagenomic data indicate an underexplored biosynthetic and ecological potential within the phylum *Acidobacteriota*. During our ongoing efforts to isolate and bioprospect bacterial diversity for natural product-related, environmental functions, we retrieved three *Acidobacteriaceae* strains from termite nest material (25) and one strain from a German soil sample (39). To broaden the diversity of analyzed strains, we included additional available representatives of this family (*Edaphobacter aggregans* DSM 19364, *Acidobacterium* sp. S8, *Silvibacterium bohemicum* S15, *Acidicapsa borealis* DSM 23886, *Granulicella rosea* DSM 18704, and *Bryocella elongata* DSM 22489) (Table S1) and performed a one-strain-many-compounds (OSMAC) approach (40), designed to explore condition-dependent metabolic diversity, potentially leading to detectable antimicrobial activity. To this end, we modified the carbon sources, vessel size, and shaking speed and applied solidification and surface-texturing methods using quartz or ceramic beads to enable cell surface attachment. Cultivations were performed on a 100 mL scale using 300 mL Erlenmeyer flasks or on a 4 mL scale using 24-well Duetz system plates (Table S2). Samples were taken on days 5, 7, 10, 12, and 14 of the respective cultivations. The generated samples were dried by lyophilization and extracted with methanol, resulting in a total of 892 organic extracts.

**Fig 1:**
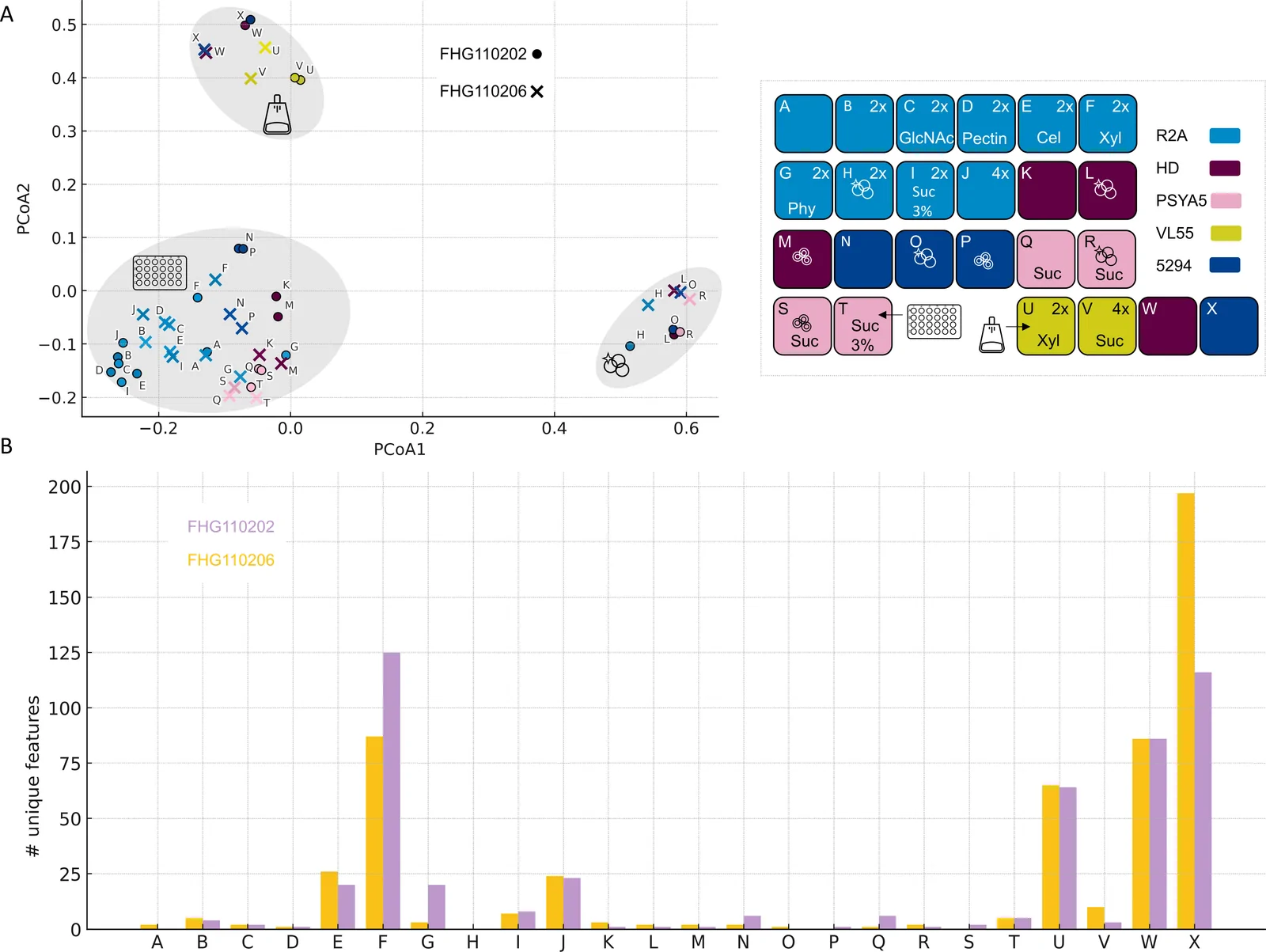
OSMAC metabolome variation by medium, strain, and cultivation scale. The legend details the medium compositions (A–X), based on base-medium color, concentration, additives, and bead status. Samples labeled A–T were cultivated in 24-well plates (small-scale), while U–X were grown in 300 mL Erlenmeyer flasks. If not stated otherwise, additives were supplied in concentrations of GlcNAc, N-acetyl-glucosamine (0.05%); Cel, cellobiose (0.5%); Xyl, xylan (0.5%); Phy, phytagel (0.8%); and Suc, sucrose (0.5%). (**A**) PCoA of control-filtered LC/MS presence/absence fingerprints using Jaccard distance. Samples are labeled by the medium letter (A–X) and colored by base-medium (see legend). Samples from FHG110202 are marked with circles, and those from FHG110206 with crosses. Light gray ellipses highlight visually distinct clusters, and icons indicate their common features: large-scale flask cultivation (flask), small-scale 24-well cultivation (24-well plate), and quartz beads containing samples (circles). (**B**) Number of unique features per medium (A–X) and strain as clustered bars. Strains are indicated by color: FHG110202 (light purple) and FHG110206 (yellow). Unique features are defined as not being present in any medium control and only present in the respective medium, but not in any other medium.

To assess the metabolic diversity of the generated extracts, we assessed the metabolic composition of two representative strains, *Acidobacterium* FHG110202 and *Terracidiphilus* sp. FHG110206 (*n* = 244, including medium controls) by performing ultra-high-performance liquid chromatography coupled to high-resolution mass spectrometry (UHPLC-QTOF-HR-MS). Molecular features (*m*/*z*, retention time) were detected and aligned into buckets, representing consensus features corresponding to the same putative metabolite across different runs. Combining time points for each strain–medium combination and presence–absence analysis led to 72 conditions (including medium controls) with 20,285 features present and distributed into 2,747 buckets (Fig. S1). To assess the effect of the medium on the strains’ metabolomes, we identified and removed medium-derived features by comparing strain extracts with uninoculated medium control extracts; features detected in the medium extracts were classified as medium-derived and excluded from further analyses (*n* = 1,795). Pairwise Jaccard distances between samples were ordinated by principal coordinates analysis (PCoA) (Fig. 1A). Metabolomic profiles clustered mainly by medium (color code) and vessel size (top, left cluster) rather than by strain. We also observed a distinct cluster for medium containing quartz beads (bottom, right cluster). A PERMANOVA analysis confirmed this observation, suggesting a pronounced condition-dependent metabolomic shift. The medium identity accounts for 87% of the variance among strain extracts (*F* = 7.09, *P* = 0.001), while the strain identity within the same medium accounts for a considerably smaller proportion (1.7%, *F* = 0.77, *P* = 0.001). Within the small-scale set, the bead condition accounted for 29.2% of the variance (*F* = 7.64, *P* = 0.001), consistent with the observed quartz-bead cluster. Thus, metabolomes are primarily influenced by the medium and vessel size, with only a small strain-level difference. Together, these results indicate that, within the analyzed data set, variation in cultivation conditions has a great influence on metabolomic profiles. This underscores the importance of employing diverse cultivation settings to comprehensively capture condition-dependent metabolic variation. When examining the number of unique metabolite features across individual cultivation conditions (Fig. 1B) (FHG110202:FHG110206), media F (87:125), U (65:64), W (86:86), and X (197:116) exhibited higher numbers of unique features compared to the average unique feature number across all media (22.2:20.7). Notably, these media correspond to large-scale (300 mL) Erlenmeyer flask cultivations, suggesting that cultivation scale, along with medium composition, is linked to a broader range of detectable metabolic features compared to small-scale (24-well plate) conditions. These observations may be attributed to differences in the (micro)environment created by various cultivation formats. Vessel size influences nutrient gradients, timing of substrate availability, surface attachment, and aeration, all of which can shape metabolic output. Larger vessels might support greater physiological diversity and more robust conditions over the rather long course of incubation. Given the oligotrophic lifestyle and slow growth of *Acidobacteriota*, this might enable the strains to persist and proliferate, thereby promoting a broader metabolic range.

To determine whether the observed increase in metabolomic diversity under different conditions led to measurable biological effects, we then tested the entire collection of OSMAC-derived extracts for antimicrobial activity. An aliquot of each extract was reconstituted in dimethyl sulfoxide (DMSO) and used to determine the antimicrobial bioactivity by screening against the bacterial indicator strains *Escherichia coli* ATCC 35218, *Bacillus subtilis* DSM 10, and *Mycobacterium smegmatis* ATCC 607, as well as the fungus *Candida albicans* FH2173. This, however, did not reveal any reproducible antimicrobial effect enabling traceability of a causative agent (data not shown).

### Result 2: genomic context of *Acidobacteriota* reveals limited biosynthetic gene cluster potential in *Acidobacteriaceae*

**Fig 2:**
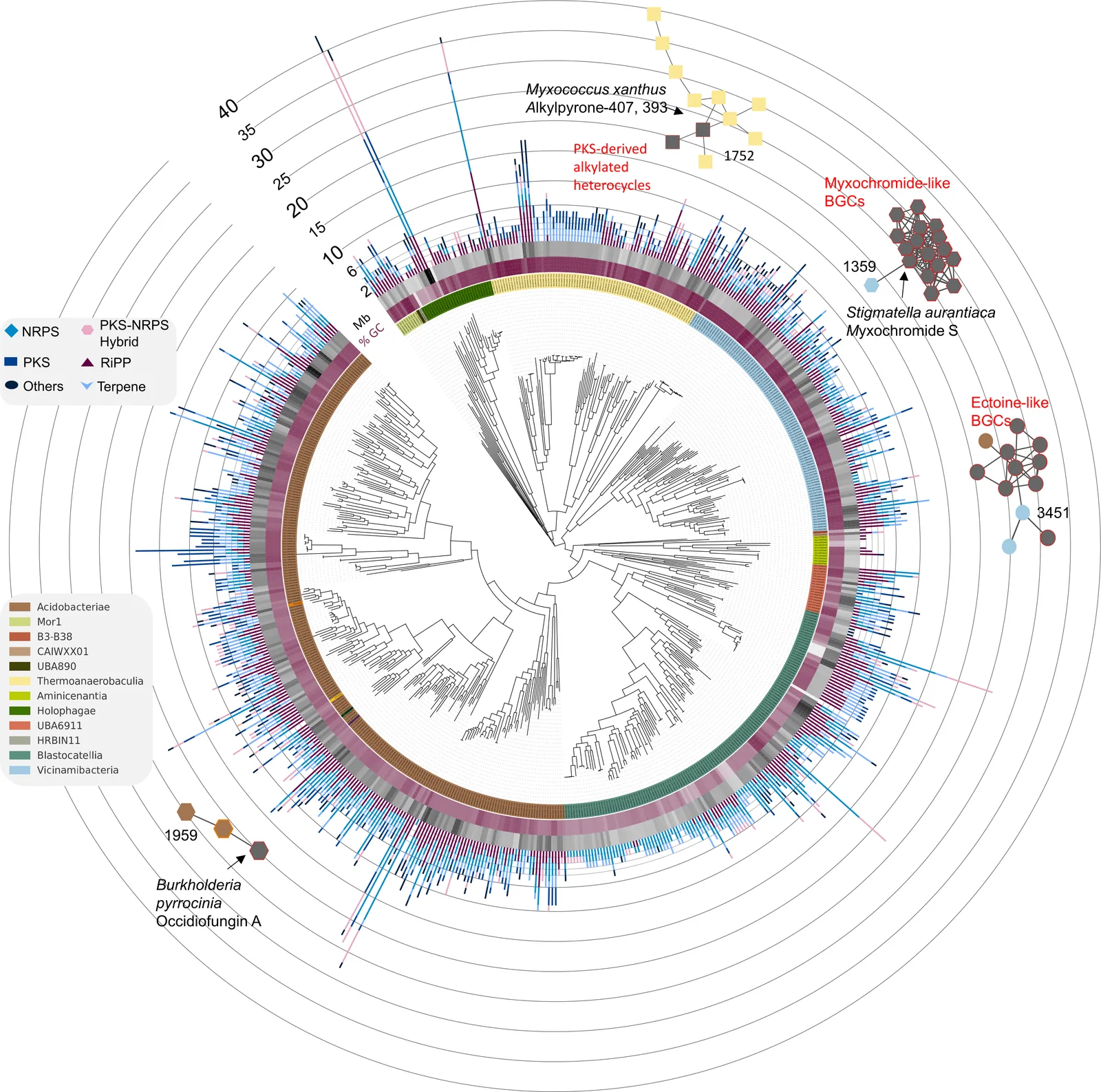
BGC composition of the Acidobacteriota phylum and BiG-SCAPE network of acidobacterial BGCs clustering with MIBiG reference clusters. Multiple-sequence alignments generated by GTDB were used to create a phylogenetic tree of 618 representatives of the Acidobacteriota. Twelve acidobacterial classes were identified by GTDB and are indicated by different colors (ring 1). FHG strains are color-coded as follows: FHG110214 (green), A. borealis DSM 23886 (purple), FHG110202 (yellow), and FHG110511 (orange). Ring two indicates the genome size of the individual representatives, ranging from black (larger genomes) to gray (smaller genomes). Bar charts indicate the total number of antiSMASH-detected BGCs, differentiated into different BGC types.

To contextualize the absence of detectable antimicrobial activity and to evaluate the biosynthetic capacity of *Acidobacteriaceae* within a broader evolutionary framework, we conducted a comparative analysis of their genomic potential within the *Acidobacteriota* phylum. For this, publicly available *Acidobacteriota* genomes (*n* = 1,428) were retrieved from the NCBI database and subjected to quality control using CheckM2 (40) (Fig. S2). By applying recognized quality criteria (completion > 90% and contamination < 5%) (41), 810 strains, including one in-house assembly (FHG110206), were disqualified from further analysis (*n* = 618). Notably, most of the analyzed genomes were retrieved from metagenomes (*n* = 533; 86%) (Table S1). Because comparative analyses of BGCs are influenced by genome assembly type, size, and GC content, these parameters were evaluated across the *Acidobacteriota* genome set. Although these analyses did not affect the primary conclusions, they offer essential contextual information and are therefore included in the supplemental material (Extended results). The taxonomic affiliation was established by GTDB (42), and the generated multiple-sequence alignment was used to build a phylogenetic tree. This confirmed the termite nest-associated strains as members of the genera *Acidobacterium* (FHG110202) and *Terracidiphilus* (FHG110214), whereas the soil-associated strain FHG110511 was identified as an *Edaphobacter*. The bioinformatically predicted features of each strain were assigned and visualized within the tree using iTOL (43). The genomes in the data set span 12 classes (Fig. 2), dominated by the *Acidobacteriae* (*n* = 242), *Blastocatellia* (*n* = 130), and *Vicinamibacteria* (*n* = 98). Notably, these classes also comprise the majority of cultured representatives within our data set, with 76 of 86 isolate genomes (88%) assigned to them. To evaluate the biosynthetic potential of the *Acidobacteriota* phylum, we predicted BGCs using the standalone version of antiSMASH (44). To evaluate potential artifacts caused by fragmented assemblies, the number of antiSMASH-predicted BGCs was compared with the number of contigs. No genomes showed a pattern indicative of inflated BGC counts associated with highly fragmented assemblies (Fig. S3).

Across the *Acidobacteriota* data set, a total of 3,865 BGCs were identified, with ribosomally produced and post-translationally modified peptides (RiPPs) being the most prevalent class (*n* = 1,152, 30%), followed by non-ribosomal peptide synthetases (NRPSs) (*n* = 978, 26%) and terpenes (*n* = 777, 13%). On average, analyzed genomes contained 6.2 ± 4.4 BGCs, although this average conceals substantial variability in BGC content across taxonomic groups. At the class level, *Acidobacteriae* exhibit the highest average BGC content, at 7.4 ± 3.7 BGCs per genome, followed by UBA6911 (6.3 ± 2.5) and Mor1 (5.8 ± 2.1). Dedicated analysis of the FHG *Acidobacteriaceae* revealed an average of 9.0 ± 3.46 BGCs, exceeding the class-level average but remaining within the range typical for the family (7.9 ± 3.0), with RiPPs, terpenes, and NRPs as the dominant BGC classes. Notably, significant deviations from class-level averages were observed at finer taxonomic resolutions. In particular, the *Holophagae* class exhibits substantial variation in BGC number (5.1 ± 10.5), indicating a higher potential among some of its representatives. This variation is primarily driven by members of the *Acanthopleuribacteraceae* family (Z-score: 3.5), which harbor more than 40 BGCs per genome—approximately seven times higher than the average for *Acidobacteriota*. Intriguingly, the first acidobacterial NPs documented to date were identified from this family, highlighting its exceptional biosynthetic potential. Collectively, these findings suggest that biosynthetic potential within *Acidobacteriota* is unevenly distributed, and that class-level averages mask lineage-specific hotspots. This highlights the importance of analyzing at the family and genus levels to identify promising targets for natural product discovery and provides genomic context for the limited antimicrobial activity observed in *Acidobacteriaceae*.

Although the total number of BGCs varied among *Acidobacteriota*, BGC counts alone do not indicate whether biosynthetic potential is distributed across lineages or restricted to specific genomic contexts. To evaluate biosynthetic conservation, lineage specificity, and overlap with known natural product gene clusters, sequence similarity relationships among all detected BGCs were analyzed. For this, we performed BiG-SCAPE analysis (45) of all 3,865 detected BGCs, including the MiBIG reference BGCs (version 3.1), applying a similarity cutoff of 0.5 (Fig. S4). The analysis revealed a highly fragmented biosynthetic landscape, in which 1,651 biosynthetic gene clusters (BGCs) remained as singletons, while only a limited number of gene cluster families (GCFs) were shared across multiple genomes (*n* = 2,212). In contrast, the largest GCF comprised only 25 members, all of which belonged to the class *Acidobacteriae*. In total, 25 GCFs were specific to a single taxonomic class, and most of these class-specific GCFs contained more than 10 members (16/25), indicating strong taxonomic conservation of certain biosynthetic traits. This taxonomic conservation is further supported by comparing the GCF composition of the different strains using cosine distance, visualized by uniform manifold approximation and projection (UMAP) (Fig. S5). Here, genomes from the same taxonomic class clustered closely, indicating overall conservation across phylogenetic groups. In contrast to these conserved patterns, the FHG *Acidobacteriaceae* did not share any clusters with one another (Fig. S6), suggesting a distinct set of BGCs covered by the OSMAC-analyzed isolates.

Together with the high number of singleton clusters, this indicates that biosynthetic diversity in *Acidobacteriota* is primarily conserved at a close phylogenetic resolution. To assess how much of this diversity correlates to characterized natural product pathways, sequence similarity relationships to MIBiG reference BGCs were subsequently examined and confirmed by CORASON alignments (45). This revealed co-clustering of seven acidobacterial BGCs across four GCFs (Fig. 2), indicating compositional BGC similarity to, e.g., the myxochromide and the occidiofungin A reference BGCs (SI–Extended results). Intriguingly, the latter is a match to our strain FHG110511, harboring a PKS/NRPS hybrid BGC that clusters in GCF1959 with the occidiofungin A reference BGC (MIBiG BGC0001711) at a pairwise identity of 70.4% (Fig. S6).

Occidiofungin A is a *Burkholderia contaminans-*produced NP that exhibits broad antifungal activity and is currently in early clinical development (gel OCF001, phase 1) for use against multidrug-resistant vaginal yeast (*Candida* spp.) infections (46, 47). A comparative evaluation of the NRPS and NRPS/PKS hybrid core gene architectures revealed a highly conserved module arrangement, with some discrepancies in predicted A-domain specificity. Testing of the strain’s organic extracts against *C. albicans*, however, showed no antifungal activity. This occidiofungin-like BGC thus represents an isolated exception within the FHG *Acidobacteriaceae* and among multimodular NRPS, PKS, or hybrid systems in this data set.

Collectively, these findings suggest that biosynthetic diversity within *Acidobacteriota* is predominantly fragmented and confined to specific lineages, with only sporadic and isolated cases exhibiting similarity to known natural product biosynthetic pathways. This genomic framework accounts for the presence of occasional biosynthetically “talented” lineages and the predominantly limited antimicrobial activity observed in the FHG *Acidobacteriaceae*.

### Result 3: dereplication and tryptophan-feeding kinetics reveal phytohormones in *Acidobacteriaceae* extracts

Given the limited potential for complex antimicrobial biosynthesis, as inferred from genomic analyses, we next investigated the chemical composition of OSMAC-derived *Acidobacteriaceae* extracts, independent of bioactivity, using untargeted metabolomics and reference-based dereplication. Using accurate mass, retention time, and MS/MS spectral matching, this analysis enabled the annotation of known metabolites in extracts from representative *Acidobacteriaceae* strains, including the phytohormones indole-3-acetic acid (IAA) and N⁶-(Δ²-isopentenyl)adenine (iP) with a confidence level 1 (identified metabolite: as proposed by the Chemical Analysis Working Group of the Metabolomics Standards Initiative) (48). In addition to more auxins and cytokinins, the dereplication workflow also covered a broader panel of phytohormones, including gibberellins, jasmonates, abscisic acid, and brassinosteroids; however, only IAA and iP were identified in the analyzed extracts.

**Fig 3:**
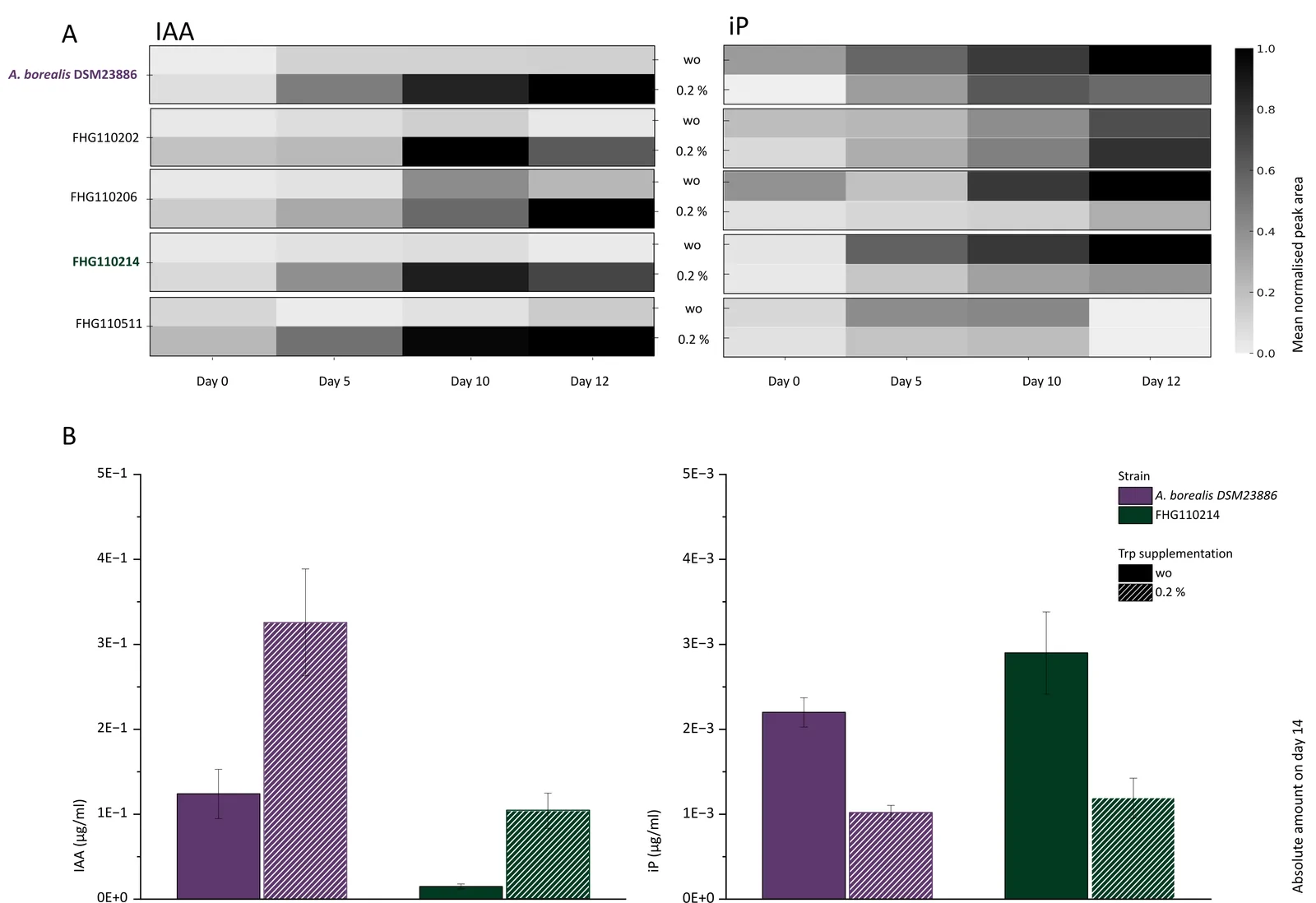
Relative quantification of IAA and iP in FHG *Acidobacteriaceae* and *A. borealis* DSM 23886 as normalized peak area (**A**), and absolute quantification of these phytohormones in the top producers FHG110214 (iP) and *A. borealis* DSM 23886 (IAA) on day 14 (**B**). (**A**) Raw peak areas were normalized against the largest peak area within one biological kinetic study for IAA and iP separately. The mean values were calculated and plotted as a heatmap to indicate temporal and dynamic induction patterns between strains and supplementation status. Trp supplementation results in higher IAA production but generally has a negative impact on iP production. This effect is less pronounced in FHG110202 and *A. borealis* DSM 23886. (**B**) Absolute concentrations were derived from a calibration curve of IAA and iP, correlating peak area with given concentrations. Cultivations were performed in triplicate, and the mean calculated concentration is shown for *A. borealis* DSM 23886 (green) and FHG110214 (purple).

While IAA production has previously been reported for members of the *Acidobacteriaceae* (31), the detection of the cytokinin iP represents a novel finding for the *Acidobacteriota* phylum. To systematically describe the cultivation conditions that favor IAA production, we visualized the peak area of IAA in FHG110202 as an example across all tested media and setups (Fig. S7). While IAA was not produced under conditions leading to the isolation of the strains (VL-55 base media), it became detectable in more complex media. Among these, R2A-based cultivation, with agitation, consistently showed the best IAA production. To further characterize the IAA and iP production of our FHG *Acidobacteriaceae* and the reference strain *A. borealis* DSM 23886, we cultivated these strains in media with and without tryptophan (Trp) supplementation, which is commonly known to increase IAA production in microorganisms (49). We performed a kinetic study to analyze the effect of 0.2% Trp on the relative production of IAA and iP over 14 days following methanolic extraction of the cultures. Overall, IAA and iP concentrations increased over time, reaching their highest levels on days 10 and 12 of growth (Fig. 3; Fig. S8). Indeed, Trp supplementation significantly enhanced IAA production in all strains. In contrast, supplementation with Trp consistently suppressed iP production in the analyzed strains, except in FHG110202 and *A. borealis* DSM 23886, where the effect was less pronounced (Fig. 3A).

This initial profiling revealed that strain FHG110214 was the most productive iP producer while exhibiting comparable IAA productivity to *A. borealis* DSM 23886, FHG110202, and FHG110206. Based on this production profile, we selected FHG110214 and *A. borealis* DSM 23886 for absolute quantification of phytohormones. When supplemented with Trp, *A. borealis* DSM 23886 produced IAA to a final concentration of 0.326 ± 0.063 µg/mL, whereas FHG110214 produced IAA at 0.104 ± 0.021 µg/mL. Instead, without supplementation with Trp, the IAA titers dropped to 0.124 ± 0.029 µg/mL (3-fold) and 0.015 ± 0.003 µg/mL (7-fold) in *A. borealis* DSM 23886 and FHG110214, respectively. This production profile is reversed for iP, where Trp supplementation resulted in decreased production for both strains FHG110214 (with Trp: 0.0058 ± 0.0012 µg/mL; without Trp: 0.0143 ± 0.0024 µg/mL) and *A. borealis* DSM 23886 (with Trp: 0.005 ± 0.0004 µg/mL; without Trp: 0.0108 ± 0.0008 µg/mL). The observed phytohormone levels align with concentrations reported for various plant-associated bacteria (50, 51), including *Streptomyces*, *Enterobacter*, and related taxa, although they are approximately one order of magnitude lower than values reported under optimized production conditions. Notably, the detected iP concentrations are consistent with those reported for bacteria such as *Achromobacter xylosoxidans* (52) and *Paenibacillus polymyxa* (53), supporting the biological plausibility of iP production by *Acidobacteriaceae*. The detection of IAA and iP suggests that the metabolic plasticity of *Acidobacteriaceae* encompasses compounds potentially involved in plant–microbe interactions. To determine whether this biosynthetic potential translates into a functional phenotype, we next evaluated the effects of phytohormone-containing extracts on barley seedling growth.

### Result 4: phytohormone-containing *Acidobacteriaceae* extracts do not promote barley seedling growth

**Fig 4:**
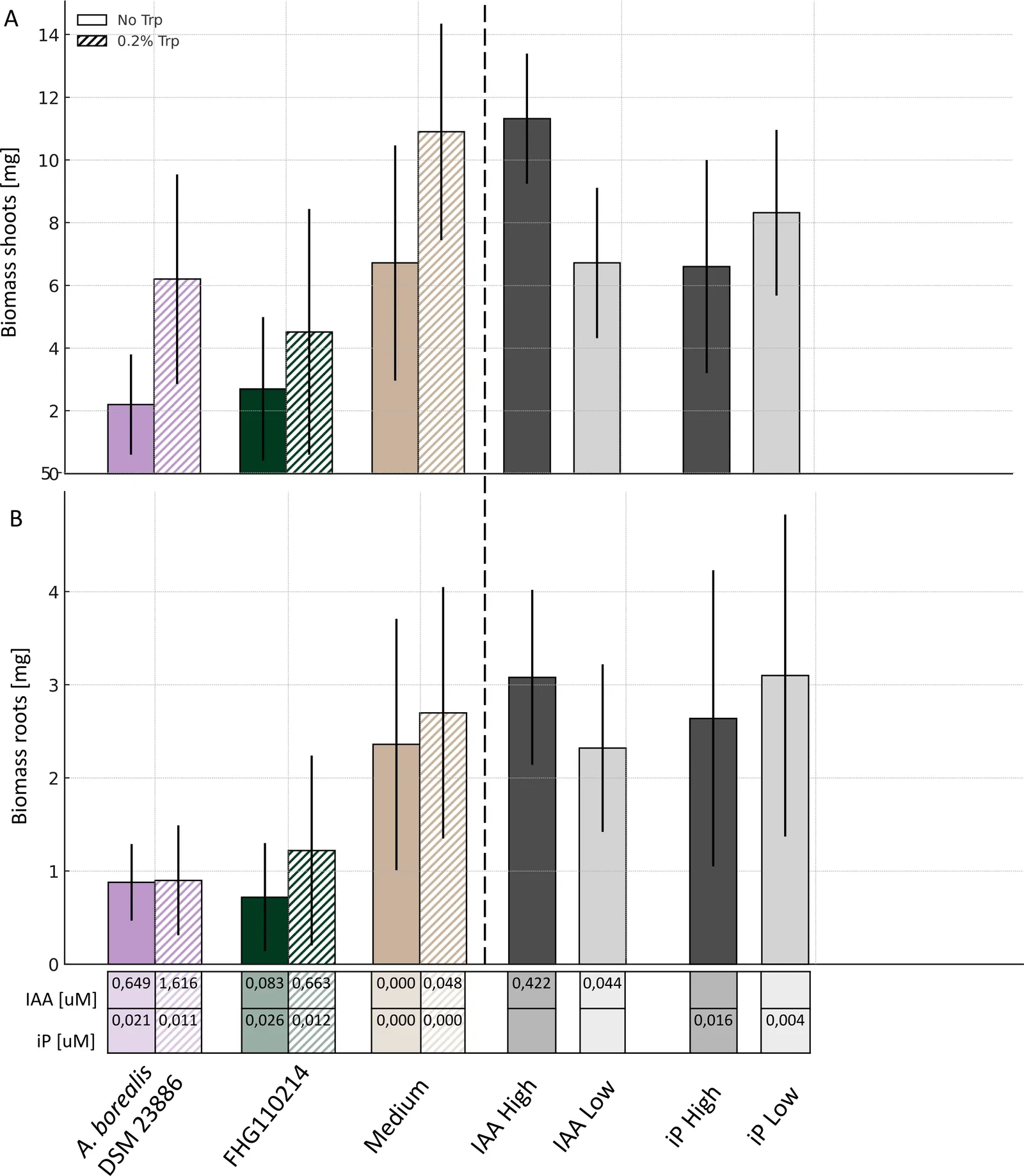
Dry biomass of barley shoots (**A**) and roots (**B**) treated with extracts of strains *A. borealis* DSM 23886 (purple) and FHG110214 (green). Striped bars indicate extracts from Trp-supplemented cultivations, while solid bars indicate that extracts were not supplemented with Trp. Medium controls (brown), as well as IAA and iP as pure compounds (gray), were also tested. The table indicates the final concentrations of phytohormone applied to the barley seedlings. In general, supplementation with Trp results in increased biomass, especially in the shoots. While pure compound controls show comparable growth to the medium control, the strain extracts inhibit plant growth. This negative effect is diminished upon supplementation with Trp, especially in the shoots.

Previous studies have shown that culture broths of IAA-producing *Acidobacteriaceae* promoted root and shoot growth *in Arabidopsis thaliana* (20, 31) and enhanced growth and chlorophyll in duckweed (54). To evaluate whether extracts containing phytohormones from *Acidobacteriaceae* exert similar effects under controlled conditions, we examined their influence on the growth of barley seedlings using cell-free extracts and defined phytohormone standards. Barley seedlings were cultivated in the presence of crude extracts derived from FHG110214 and *A. borealis* DSM 23886 (1-fold final concentration), alongside medium controls and pure compound standards (Fig. 4; Fig. S9). Throughout the treatments, Trp supplementation generally increased biomass compared to unsupplemented controls, although this trend was more pronounced for shoot biomass and was weaker or not consistently observed for root biomass across all strains (Fig. 4A and B). In contrast, crude extracts from FHG110214 and *A. borealis* DSM 23886 did not promote barley seedling growth. Instead, both shoot and root biomass were reduced relative to controls, irrespective of whether extracts originated from Trp-supplemented or unsupplemented cultures (Fig. 4A and B). Notably, Trp supplementation during bacterial cultivation partially mitigated the inhibitory effect of the extracts, suggesting that cultivation conditions influence extract composition beyond phytohormone content alone.

These findings differ from previous reports that demonstrated plant growth promotion by *Acidobacteriaceae* using live bacterial cells or culture broths in direct contact with plant roots (31). The present experimental design utilized cell-free organic extracts to ensure a defined and controlled input; however, this method may not replicate localized, high-concentration phytohormone gradients or other growth-promoting characteristics found in living systems. In natural rhizosphere environments, bacterial cells or cell-free supernatants may supply enzymes, signaling molecules, or additional metabolites that interact synergistically with phytohormones, effects not captured by organic extracts alone.

Interestingly, our findings, along with those of Kielak et al. (31) and Gonçalves et al. (20), suggest that phytohormone production is common among *Acidobacteriota*, particularly among strains isolated from decaying wood or lignocellulosic substrates. This raises intriguing questions about the ecological roles of *Acidobacteriota* strains producing compounds such as IAA and iP in natural systems (55). Taken together, these results confirm that *Acidobacteriaceae* produce phytohormones in relevant amounts, but their role in PGP remains uncertain.

### Result 5: genomic context of phytohormone production and plant growth-promoting traits in *Acidobacteriota*

Given the absence of detectable plant growth-promoting effects despite phytohormone production, we next asked whether this outcome is consistent with the genomic repertoire of *Acidobacteriaceae*. To address this, we examined the distribution of plant growth-promoting traits (PGPTs) across the *Acidobacteriota* phylum and analyzed pathway-specific genomic determinants underlying phytohormone biosynthesis in *Acidobacteriaceae*.

#### Global distribution of PGPTs across *Acidobacteriota*

**Fig 5:**
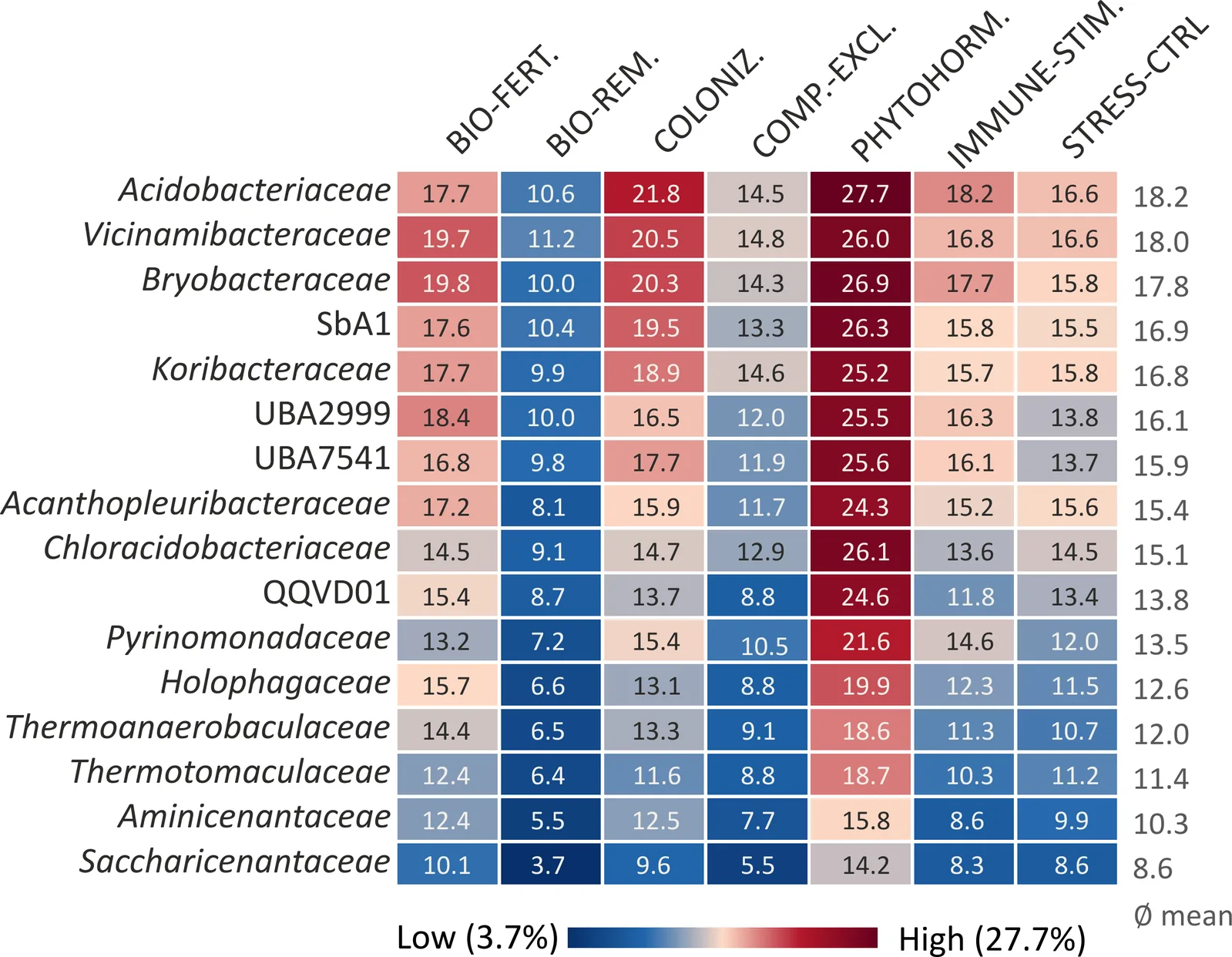
PGPT family coverage across *Acidobacteriota* families. The heatmap shows the mean percentage of PGPT families detected within PLABASE functional categories (level 2) for *Acidobacteriota* families with formal taxonomic names or more than 20 available genomes (*n* = 16). PGPT coverage values represent the proportion of PGPT families detected per functional category relative to the total number of PGPT families defined for that category in the PLABASE ontology. Families (rows) are ordered by their overall mean percentage coverage (right column) across the seven PGPT categories, from lower to higher functional coverage. Columns correspond to the seven PGPT categories analyzed. Abbreviations: BIO-FERT., bio-fertilization; BIO-REM., bio-remediation; COLONIZ., colonizing plant system; COMP.-EXCL., competitive exclusion; PHYTOHORM., phytohormone and plant signal production; IMMUNE-STIM., plant immune response stimulation; STRESS-CTRL., stress control and biocontrol. Overall, phytohormone and plant signal production displayed the highest representation across families, whereas bioremediation showed the lowest coverage.

Using a DIAMOND-based workflow against the PLABASE database, we analyzed 618 curated *Acidobacteriota* genomes to assess PGPT coverage and identify biosynthetic pathways potentially supporting IAA and cytokinin production. For family-level comparisons, only families with formal taxonomic descriptions or ≥20 representative genomes were considered (*n* = 391; 64.2%). PGPT family coverage was aggregated at the GTDB-Tk family level and clustered by PLABASE level-two categories (Fig. 5).

To facilitate interpretation of PGPT-associated functional coverage, the families were ordered in the heatmap according to their overall mean percentage coverage across the seven PGPT categories. Based on this ranking, *Acidobacteriaceae* showed the highest overall PGPT coverage (18.16%), followed by *Vicinamibacteraceae* (17.95%), *Bryobacteraceae* (17.83%), *SbA1* (16.91%), and *Koribacteraceae* (16.82%). UBA2999 (16.07%) and UBA7541 (15.95%) also ranked among the more highly equipped families overall, whereas QQVD01 showed an intermediate overall coverage (13.80%). Across all families, the phytohormone/plant signal production category consistently showed the highest coverage, whereas bioremediation was the least represented. *Acidobacteriaceae* was among the most functionally enriched families, with particularly high coverage in the colonizing plant system category and in phytohormone/plant signal production.

#### Genomic determinants of IAA biosynthesis

We next examined DIAMOND outputs for enzymatic equipment supporting IAA production in FHG *Acidobacteriaceae* strains. In bacteria, IAA biosynthesis proceeds through three main tryptophan-dependent routes: the indole-3-pyruvic acid (IPyA), indole-3-acetamide (IAM), and tryptamine (TAM) pathways (49). These pathways differ mainly in their intermediate compounds but converge at the formation of IAA. All FHG *Acidobacteriaceae* genomes encode the core Trp biosynthesis genes (trpA-E) and terminal enzymes for IAA production, such as an *amiE* homolog for the hydrolysis of indole-3-acetamide, and two aldehyde-dehydrogenases that may catalyze the final step of IAA production in both the TAM and the IPyA pathways. However, intermediate enzymes specific to the indole-3-pyruvic acid , indole-3-acetamide , or tryptamine pathways were not identified by the automated setup. Targeted LC-MS with authentic standards identified indole-pyruvic acid (CAS: 392-12-1) upon Trp supplementation, whereas tryptamine (CAS: 61-54-1) was not detected in FHG110202, FHG110214, and *A. borealis* DSM 23886. FHG110511 showed neither intermediate. These observations support IPyA as the predominant route over TAM, while IAM remains a possible but unconfirmed option.

#### Cytokinin biosynthesis pathways and Trp responsiveness

The production of iP can arise by two distinct pathways: a widespread tRNA-isopentenyltransferase (*miaA*)-dependent pathway that leads to the context-dependent formation of iP through the degradation of these tRNAs (56–58) and a less common adenylate IPT/LOG-dependent pathway associated with regulated cytokinin output in bacteria (59–61). Genomic analysis revealed that FHG110511, FHG110214, and *A. borealis* DSM 23886 encode *miaA* and associated accessory genes, whereas only FHG110202 additionally harbors a LOG enzyme. No adenylate IPT homologs were detected in any FHG *Acidobacteriaceae* genome. The observed Trp-dependent reduction in iP production in strains encoding only the tRNA-associated pathway may reflect altered tRNA turnover under high Trp availability. Increased intracellular Trp levels may reduce the need for amino acid biosynthesis and associated tRNA turnover, thereby limiting the release of iP from prenylated tRNAs. In contrast, FHG110202, the only analyzed strain with a LOG enzyme, did not show a Trp-dependent decrease in iP production. This suggests that LOG-associated cytokinin activation may decouple iP levels from global translation dynamics and tRNA turnover.

#### Absence of key functional PGPTs in FHG *Acidobacteriaceae*

Several plant growth-promoting traits are routinely assessed in genome-based surveys and classical wet-lab assays. We next examined whether the absence of experimentally tractable PGPTs could explain the lack of plant growth promotion observed in our bioassays. These traits are well-established mechanisms of plant growth promotion. Phosphate solubilization increases the availability of inorganic phosphate to plants. Nitrogen fixation supplies biologically accessible nitrogen, and ACC deaminase activity reduces plant ethylene levels by degrading 1-aminocyclopropane-1-carboxylic acid, which alleviates stress-induced growth inhibition. No *acdS* homologs were detected in the FHG *Acidobacteriaceae*. The enzyme AcdS, assigned to PGPT family 7345, was also searched for across the entire *Acidobacteriota* data set but was not detected in any strain. Genes linked to phytate-derived phosphate solubilization, including *appA*, were found in *A. borealis* DSM 23886, FHG110214, and FHG110511, as well as in 117 genomes within the entire data set, but only among *Acidobacteriae* and *Blastocatellia*. Nitrogen fixation gene clusters (*nifA–F*) were identified in selected *Holophagaceae* and *Bryobacteraceae* genomes, but not in any FHG *Acidobacteriaceae* strain. This is in accordance with a recent study by Gonçalves et al. (20) describing the presence of *nif* clusters in members of the *Holophagaceae* and *Acidobacteriaceae* families.

#### Comparison with previous PGPT surveys

Compared to the large-scale metagenomic analysis of Gonçalves et al., our PGPT analysis shows both shared and divergent trends across the acidobacterial families. Some families, such as *Acidobacteriaceae*, *Thermoanaerobaculum*, *Aminicenantesaceae*, *Holophagaceae*, and *Koribacteraceae*, are grouped similarly in terms of their overall equipment, either uniformly high or low; other families show significant discrepancies. Specifically, our results diverge for the families *Bryobacteriaceae*, *Vicinamibacteraceae*, *Pyrinomonadaceae*, and *Chloracidobacteriaceae*, where the PGP potential differs substantially. These differences likely arise from the limited overlap in representative genomes between the two datasets. Overall, 9.2% of our data set is also covered by the Gonçalves data set. Additionally, our analysis includes seven additional *Acidobacteriota* families. Notably, the data set coverage is particularly low for most families with detected discrepancies, including *Bryobacteriaceae* (1/74), *Vicinamibacteraceae* (2/6), and *Pyrinomonadaceae* (2/87). The overlap of the *Chloracidobacteriaceae* (10/14) is relatively high; however, in addition to the data set composition, methodological differences likely also contribute to the differences. Gonçalves et al. focused on 91 curated PGPTs, whereas our genome-mining approach utilized the complete PLABASE database, encompassing more than 1,300 plant-growth-promoting gene families. This broader functional scope may capture additional trait diversity and help explain the differential PGPT evaluation and distribution observed across families. Importantly, PGPT profiles of *Acidobacteriaceae* were highly consistent across both studies, showing a conserved enrichment of phytohormone-related traits and phosphate-associated functions.

Taken together, these results indicate that *Acidobacteriaceae* harbor a conserved and comparatively rich repertoire of plant-associated traits, including phytohormone biosynthesis, but lack key functional determinants commonly linked to direct plant growth promotion. This genomic configuration aligns with the limited growth-promoting effects observed in cell-free assays and suggests that *Acidobacteriaceae*-mediated plant interactions are likely context-dependent and multifactorial.

### Result 6: tryptophan induces metabolomic reprogramming and antifungal activity in *Acidobacteriaceae*

**Fig 6:**
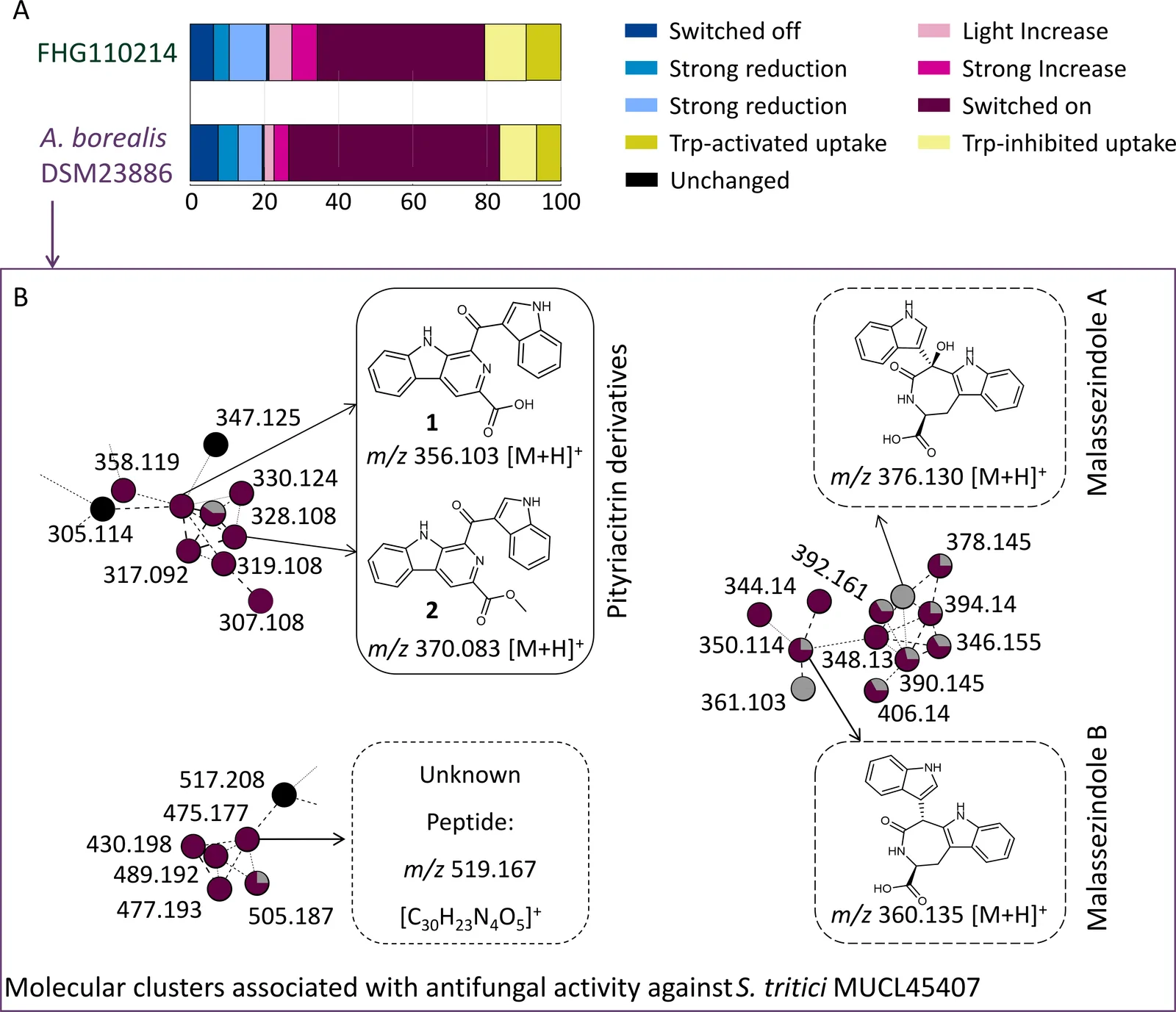
Overall metabolic response of cultivation with Trp in strains FHG110214 and *A. borealis* DSM 23886. (**A**) Bar charts illustrating the response of metabolite features to tryptophan (Trp) supplementation. Feature presence in cultures grown with and without Trp was compared to classify their behavior. Features detected exclusively under Trp-supplemented conditions are defined as “switched-on” (dark red), whereas features absent upon Trp supplementation are classified as “switched-off” (dark blue). Signals corresponding to medium components that were differentially metabolized in response to Trp are shown in yellow. (**B**) Molecular network of MS/MS data obtained from tryptophan-induced *A. borealis* DSM 23886 extracts following µFrac-coupled bioactivity screening against *S. tritici* MUCL 45407. The network is shown as a reduced representation, including nodes corresponding to features directly associated with antifungal activity or clustering with activity-linked features. Active masses were exclusively detected (“switched on”) upon tryptophan supplementation. Several features remained unannotated (e.g., *m*/*z* 519.167), whereas others were dereplicated based on MS/MS comparison with reference data (e.g., malassezindoles A and B) or structurally elucidated after isolation (pityriacitrin B and its methyl ester).

Tryptophan is a common precursor for indole-derived metabolites and is abundant in plant-associated environments (62, 63). To determine whether tryptophan supplementation induces broader metabolic reprogramming in *Acidobacteriaceae* and results in the production of bioactive compounds, we conducted time-resolved untargeted metabolomics analyses of cultures cultivated with and without tryptophan supplementation. Ultra-high-performance liquid chromatography–quadrupole time-of-flight high-resolution mass spectrometry (UHPLC-QTOF-HRMS) and subsequent principal component analysis (PCA) of log2-transformed feature intensities (Fig. S10) revealed that the samples did not exhibit a strict separation by strain or cultivation day, but instead were separated by supplementation status, indicating a broad metabolomic shift associated with Trp supplementation. This effect was particularly evident for *A. borealis* DSM 23886 and increased over time in Trp-supplemented cultures. A quantitative comparison of detected features revealed a marked increase in metabolomic complexity upon Trp supplementation. For *A. borealis* DSM 23886, the number of detected features increased from 165 to 684 (4.1-fold), while FHG110214 exhibited an increase from 109 to 337 features (3.1-fold) (Fig. S11, X1 samples, top). This trend was independently confirmed in a second cultivation experiment (Fig. S11, X2 samples, bottom). Across both strains, approximately 80% of features present under both conditions were upregulated in Trp-supplemented cultures, whereas only 19% were downregulated and 1% remained unchanged. To further investigate Trp-dependent metabolomic responses, we categorized features based on presence–absence and fold change behavior, indicating whether features were exclusively present or absent (switched on vs switched off), or whether the production altered upon Trp supplementation (Fig. 6, top bar chart). The “switched on” class accounts for the largest fraction in both strains (*A. borealis* DSM 23886: 56.8%; FHG110214: 45.1%). Specifically, 10.4 and 16.9% of features exhibit an “increased production” profile, while 7.5 and 6.3%, respectively, are “switched off.” Small proportions (below 10%) represent different metabolization patterns of medium components under induced and uninduced conditions (Trp activated or inhibited uptake).

Given the pronounced metabolic changes observed in Trp-induced extracts, we were encouraged to re-enter our initial bioactivity discovery approach, now particularly testing the generated extracts from FHG110214 and *A. borealis* DSM 23886 for activity against the phytopathogenic fungus *Septoria tritici* MUCL 45407, a residue-borne wheat pathogen causing *Septoria tritici* blotch (STB), one of the most significant diseases of wheat (64). By testing our extracts in a microbroth dilution assay, we indeed determined a strong growth-inhibitory effect exclusively in the samples that underwent Trp supplementation. For the identification of activity-causing compounds, one representative active extract of FHG110214 and *A. borealis* DSM 23886 was microfractionated and re-screened against *S. tritici* MUCL 45407. Based on the molecular formula and the observed MS/MS fragmentation pattern, several Trp-related compounds were dereplicated within the active fractions of both strains, with a confidence level 2 (putatively annotated metabolites (48)) (Fig. 6B).

This enabled the annotation of hydroxyglyantrypine (65), as well as malassezindoles A and B (66), and putative, yet undescribed derivatives thereof (*m*/*z*: 148.1349, 376.1293, 360.1348, and 362.1498) (Fig. 6). Notably, malassezindole production is known to be strongly dependent on Trp as the sole nitrogen source in their fungal production strains (67). This observation aligns well with our cultivation conditions and experimental setup.

Besides malassezindoles, we annotated the β-carbolines acetyl-beta-carboline, pityriacitrin B (66, 68, 69), and a methylated derivative thereof. Pityriacitrins are produced by fungi and bacteria (70) and are recognized for their UV-protective properties (71). Furthermore, the β-carbolines scaffold has been recently reviewed in the context of antifungal activity (72). Indeed, pityriacitrin A, B, and their methyl esters have been described to inhibit fungal growth (70, 73). Furthermore, pityriacitrin B was shown to have protective activity against fungal colonization of plant hosts (70). In bacterial systems, beta-carboline cores, such as those found in pityriacitrin, are formed via Pictet-Spenglerase enzymes (74–76). Instead, biosynthesis of the Trp-dependent metabolites closely related to the malassezindoles has been described for the smut fungus *Ustilago maydis*, where a single enzymatic reaction, catalyzed by the enzyme Tam1, is essential for their formation, converting Trp to indolepyruvate (77). Although the biosynthetic pathways for these compounds have not been elucidated in bacteria, a similar mechanism can be inferred. However, a BLAST search for analogous proteins within our acidobacterial genome data set did not yield any significant hits. An additional signal observed in the active fraction, co-eluting with malassezindoles under the applied chromatographic conditions, suggested the presence of a peptide, with an *m*/*z* value of 519.1655 [M + H]^+^, corresponding to the molecular formula C_30_H_22_N_4_O_5_.

We focused our isolation efforts on compounds previously reported to exhibit antifungal properties. Preparative and semi-preparative isolation methods were employed on the fractions containing pityriacitrin and its derivative. Two compounds were purified, and their structures were elucidated by NMR analysis in DMSO-*d*_6_, employing ^1^H, ^13^C, COSY, HSQC, HMBC, TOCSY, ROESY, and NOESY measurements. Compound **1** (C_21_H_13_N_3_O_3_) was identified as pityriacitrin B, while compound **2** (C_22_H_15_N_3_O_3_) was determined to be its methyl ester. The spectroscopic data for both compounds were consistent with literature reports (69, 78). Detailed ^1^H and ^13^C NMR data, as well as key COSY, ROESY, and HMBC correlations, are summarized in the Supporting Information (Tables S3 and S4).

Taken together, our results demonstrate that members of the *Acidobacteriaceae* exhibit pronounced metabolic plasticity in response to cultivation conditions, especially following tryptophan supplementation, which induces extensive metabolomic reprogramming and facilitates the production of bioactive metabolites. While genomic analyses suggest a modest and uneven distribution of complex biosynthetic gene clusters within this lineage, both untargeted metabolomics and microfractionation-based bioactivity screening demonstrate that *Acidobacteriaceae* can access a chemically diverse and functionally relevant metabolite repertoire upon specific environmental cues. However, the presence of phytohormones did not translate into consistent plant growth-promoting effects in our simplified, cell-free assays setup. Genome mining offers one possible explanation for this observation, indicating that *Acidobacteriaceae* possess a conserved though incomplete set of plant growth-promoting traits. Genes related to phytohormone biosynthesis and phosphate mobilization are prevalent. Instead, essential determinants typically associated with direct plant growth promotion, such as ACC deaminase activity or nitrogen fixation, are either absent or unevenly distributed. This genomic profile indicates that acidobacterial contributions to plant–microbe interactions are likely context-dependent and may depend on spatial proximity and synergistic effectors interfacing small metabolites, EPS, and enzymes, the latter two of which are not represented in organic extract-based assays.

Our genome-mining survey of 618 high-quality *Acidobacteriota* genomes offers a complementary perspective to the large-scale MAG-based analysis by Gonçalves et al. (20), which analyzed 758 MAGs. Both studies identify *Acidobacteriaceae*, *Bryobacteriaceae*, and *Koribacteraceae* as families with enriched PGPT potential, while families such as *Holophagaceae* and *Vicinamibacteraceae* appear comparatively under-equipped. However, notable discrepancies emerged, possibly reflecting differences in the underlying data sets and the methodological approach. The Trp-dependent induction of antifungal activity and the identification of indole-derived metabolites, such as β-carbolines and malassezindole-related compounds, reveal a previously underestimated capacity of *Acidobacteriaceae* to produce bioactive secondary metabolites. The observation that bioactivity is restricted to Trp-supplemented cultures emphasizes the impact of environmental signals in triggering metabolic responses. The phenotype indicates that precursor availability in the natural ecosystems—the Trp-enriched plant environment—may be a primary factor shaping the functional output of these bacteria (79).

Despite the current technical limitations, exploring *Acidobacteriota* as a source of novel bioactive compounds or as contributors to plant–soil health remains an intriguing avenue. A considerable limitation, however, is their challenging growth in laboratory settings. If obtained as isolates, their total cell biomass remained low, even when using optimized medium conditions. Some strains also show aggregated growth forms, further complicating their handling in the lab. Given the intriguing biosynthetic potential predicted from the genomic data, more efforts are needed to generate know-how in characterizing and exploiting this phylum. This requires, for example, the development of genetic tools for *in situ* manipulation, overexpression, and deletion of specific genes, as well as the establishment of a genetically tractable heterologous expression system in *Acidobacteriota*-hosts. Together, this could lead to a better understanding and utilization of *Acidobacteriota*, thereby establishing a foundation for future research into their potential functions in the environment as well as for agriculture, bioremediation, and natural product discovery.

## MATERIALS AND METHODS

### Microbial cultivation of acidobacterial strains

#### Routine cultivation of *Acidobacteriota*

Acidobacterial strains were routinely cultivated in R2A medium (R2A broth, HIMEDIA, Maharashtra, India) buffered with MES-hydrate [2-(N-morpholino)ethanesulfonic acid hydrate, CAS: 1266615-59-1] (1.95 g/L) at pH 6.0. Cultures were incubated at 28°C with shaking at 75 rpm. To sustain healthy and continuous growth, 10 mL of a 7-day-old preculture (30 mL culture volume in a 100 mL Erlenmeyer flask) was used to inoculate 90 mL of fresh medium in 300 mL Erlenmeyer flasks, resulting in a final culture volume of 100 mL and an inoculation ratio of 10% (vol/vol). For quality control, we transferred 100 μL after each inoculation or sampling step to an R2A-MES-buffered agar plate to observe colony morphologies and identify the growth of contaminant microorganisms. Additionally, we performed 16S rRNA gene amplification testing with primer pair E8F (50-GAG TTT GAT CCT GGC TCA G-30) and 1492R (50-ACG GYT ACC TTG TTA CGA CTT-30) (80).

#### Cultivation strategies for OSMAC screening and tryptophan-induced metabolomics

For the OSMAC cultivation approach, we performed 24-well-scale and Erlenmeyer flask-scale cultivation. We prepared forty 24-well Duetz system plates with 20 different media (each medium 4 mL) and autoclaved the plates before inoculation (10%) with a preculture grown for 14 days. Applied media were diluted medium 5294 (81) (2 g soluble starch, 2 g glycerol, 0.5 g corn steep liquor, 1 g peptone, 0.4 g yeast extract, 0.2 g NaCl; pH 5.5, buffered with 1.95 g/L MES), HD (0.5 g casein peptone, 0.1 g glucose, 0.25 g yeast extract; pH 5.0, unbuffered; DSMZ ID 1135), and PSYA 5 (26) (1.8 g KH_2_PO_4_, 0.2 g MgSO_4_ × 7H_2_O, 1 g yeast extract; optionally with the addition of sucrose at 0.5% or 3%; pH 5.0, unbuffered), as well as a diversity of VL55-based and R2A-based (1-fold, 2-fold, 4-fold) media generated by the optional addition of different carbon sources (pectin, GlcNAc – 0.05%; xylan, cellobiose – 0.5%; and sucrose 3%; applied to 2-fold R2A) (Table S2) (25, 39). Furthermore, as a surface attachment-enabling strategy, we added ceramic beads (hollow cylinders; three pieces/well plus 4 mL of media), quartz beads (round; 10 pieces/well plus 4 mL of media), or solidified the respective medium using 0.8% phytagel. The plates were incubated at 28°C, shaking at 120 rpm or with manual shaking once per day. Cultivation in a 300 mL Erlenmeyer flask was executed in 100 mL of medium by inoculation (10%) with a preculture grown for 7 days. The cultures were incubated at 28°C for 14 days with or without shaking at 75 rpm. We added 20 g of ceramic beads (Eheim mech) to non-shaking cultures. After cultivation, cultures were frozen, freeze-dried, and extracted (see below). For Trp-induced kinetic studies, we used a 1.2-fold concentrated R2A medium buffered with 2-(*N*-morpholino)ethanesulfonic acid (MES) at a pH of 6.0, which was supplemented in a 4:1 ratio with 1% sterile-filtered Trp (CAS 73-22-3) to a final concentration of 0.2% Trp and a 1-fold concentrated medium.

All kinetic (OSMAC and Trp induction) studies were performed in 300 mL flasks, with at least duplicate samples. For kinetic sample collection on days 5, 7, 10, and 12, we transferred 4 mL of culture to 24-deep-well plates for further processing. After the final harvest day, the 24-well plates were freeze-dried and extracted using methanol (SI-1.1). Since the malassezindoles have not yet been described in bacteria, we included expanded quality control steps within the respective experiments, consisting of routine agar plating performed concurrently with sampling at the beginning and end of cultivation. Therefore, culture samples were plated on agar (R2A-MES, pH 6.0) and used for 16S rRNA and 18S rRNA gene sequencing. Agar plates were incubated for at least 14 days, documented, and routinely inspected using a binocular microscope. Neither measure raised any concerns about handling fungal-contaminated samples.

### Bioassays

#### Antimicrobial screenings

Antimicrobial screenings of crude extracts were performed using microplate broth dilution assays at the final assay concentration of 0.25-fold, 0.5-fold, 1-fold, and 2-fold (in relation to the culture volume) against bacteria and fungi (*Escherichia coli* ATCC 35218, *Bacillus subtilis* DSM 10, *Mycobacterium smegmatis* ATCC 607, *Candida albicans* FH 2173, and *Septoria tritici* MUCL 45407), as described previously (39). Crude extracts showing at least 70% growth inhibition were considered bioactive and were subjected to microfractionation for dereplication of bioactivity-causing metabolites (39).

#### Barley assays

Barley seedlings (Organic Grains “Summer Barley” [EAN 7640126481333] from Sativa Rheinau purchased at Samenhaus Müller GmbH) were first disinfected and treated with antibiotics to prevent the growth of other competing microorganisms. To do this, commercially available bleach (DanKlorix) was diluted with water to a final concentration of 25%. The required number of barley seeds was selected and transferred into a beaker. Seeds were covered with bleach solution and incubated for 15 min with continuous stirring. Afterward, the seeds were rinsed five times with distilled water. Seeds that were floating in the water were removed from the sample. Finally, seeds were dried under a sterile bench on paper towels for 5 h before being stored in a sterile 50 mL Falcon tube. For plant testing, sterile glass vials (Fisher Brand, Cat. No. 14-961-34) were filled with 5 mL of SH medium (Schenk and Hildebrandt basal salt mixture, 3.2 g/L) and (pH 5.8) containing 0.25% Gelzan (Gelrite) for solidification. The medium was then further supplemented with Pen/Strep Solution (final concentration: 1 U/µL penicillin and 1 μg/mL streptomycin) and nystatin (final concentration: 0.64 μg/mL) to prevent bacterial and fungal growth. After solvent evaporation, 50 µL of 100×-concentrated microbial extracts were spotted onto the medium. After further evaporation, the barley seeds were transferred to the medium and covered with a sterile plastic cover (Kuble Art. No. 73660-25, 25 mm). Vials with seeds were incubated in the growth chamber for 72 h in the dark before switching to a day and night cycle with intervals of 16 h light and 8 h dark at 24°C and 18°C, respectively. We grew seeds for 6 days before the final evaluation with random positioning every day. For the final assessment, the vials were boiled to liquefy the medium, the medium was washed off the roots, the roots were separated from the seedlings, and the samples were lyophilized to determine the dry weight of the different plant components.

### Creation and curation of the *Acidobacteriota* genome data set

As of March 2023, *Acidobacteriota* assemblies matching our selection criteria (NCBI: txid57723; filter: RefSeq annotation) were retrieved from NCBI in .gbff file format. In addition, four *Acidobacteriota* assemblies from strains isolated from termite nest material (FHG110202, FHG110214), soil (FHG110511), and a genome generated for *A. borealis* DSM 23886 complemented the data set (*n* = 1,425). Genome assemblies were selected based on their completeness and contamination using CheckM2 (82). Only high-quality genome assemblies defined by MIMAG (41) evaluation criteria, demonstrating 90–100% completeness and 0–5% contamination, were retained for further evaluation (*n* = 648). For this, redundancies and taxonomic affiliation were checked by GTDB-Tk v2.2.6 with the corresponding GTDB reference database release R207 (42). In total, 618 curated genome assemblies were processed for metadata evaluation, BGC potential, and PGPT mining (SI–Extended methods and results for details). To correlate genomic data with taxonomic affiliation, the concatenated multiple-sequence alignment of conserved marker genes generated by GTDB-Tk was exported and used for phylogenetic reconstruction. Phylogenetic inference was performed using FastTree v2.1.5 under default parameters for amino acid alignments. The resulting tree was visualized using iTOL v4 (43).

### Analyses of the genome data set

#### Standard approaches

Nucleotide sequence alignments were generated using ClustalW v2.1 implemented in Geneious under default parameters (gap opening penalty = 15; gap extension penalty = 6.66).

#### BGC similarity

BGCs predicted by antiSMASH were grouped into GCFs using BiG-SCAPE v1.1.2, including MIBiG version 3.1. To assess whether genome fragmentation might bias biosynthetic gene cluster (BGC) detection, the number of antiSMASH-predicted BGCs was plotted against the contig number of each genome assembly (Fig. S3). The BiG-SCAPE output tables were parsed using a custom Python script (Python 3.9) to construct a binary matrix describing the presence or absence of each GCF per genome. Pairwise similarities between genomes were calculated using cosine similarity implemented in the scikit-learn library. The resulting strain-by-strain similarity matrix was projected into two dimensions using uniform manifold approximation and projection (UMAP) implemented in the umap-learn package with the cosine metric and a fixed random seed (random_state = 42). The two-dimensional embedding was visualized using matplotlib, with genomes colored according to their taxonomic classification.

#### Genomic analysis of PGPT traits

The coding sequences from the previously described genomic data set were extracted using a custom Python pipeline built with BioPython (SeqIO). To generate FASTA documents for further processing, we retrieved the gene name, product description, and translated protein sequence for each CDS. For pre-existing FASTA files, the extraction of CDSs can be skipped. To document the number of CDSs extracted per genome, a log file was generated. The PLABASE PGPT database (83) (PGPT_BASE_nr_Aug2021n_ul_1.dmnd) was used as a reference for a similarity search against the extracted protein sequences using DIAMOND v2. The script supports various sensitivity modes, enabling users to select the most suitable mode based on their specific input. The parameters and applied evaluation thresholds used in this study are described in the “Extended methods” section of the Supplemental material.

### Mass spectrometry

Ultra-high-performance liquid chromatography (UHPLC) coupled with quadrupole time-of-flight (QTOF) high-resolution mass spectrometry (HR-MS) and tandem MS (MS/MS) was performed using an Agilent 1290 Infinity LC system connected to a maXis II QTOF mass spectrometer (Bruker Daltonics) equipped with an electrospray ionization (ESI) source. Separation of samples was achieved on an Acquity UPLC BEH C18 column (130 Å pore size, 1.7 µm particle size, 2.1 × 100 mm) fitted with a matching pre-column (2.1 × 5 mm, 1.7 µm, 130 Å) at a temperature of 45°C. Chromatographic separation was achieved using a linear gradient elution of solvent A (water with 0.1% formic acid) and solvent B (acetonitrile with 0.1% formic acid) at a constant flow rate of 0.6 mL/min. The gradient program was as follows: 95% A at 0.00–0.30 min, gradually decreasing to 4.75% A by 18.00 min, then to 0% A at 18.10 min, held until 22.50 min, returned to 95% A at 22.60 min, and equilibrated until 25.00 min. Mass spectra were recorded in positive mode over a mass range of *m*/*z* 50–2,000 with a spectra rate of 1 Hz. MS/MS acquisition was performed at a 6 Hz scan rate, targeting the top five most intense ions per full mass spectrum for collision-induced dissociation (CID) using nitrogen, as per the table published by Eichberg et al. (84). Precursors were excluded after two spectra, with a release duration of 0.5 min, and re-evaluated if their signal intensity increased by ≥1.5-fold. All data analysis was performed with DataAnalysis 4.4 (Bruker, Billerica, MA, USA).

#### Chemotype-barcoding matrix

Raw data processing and data management were performed as previously published (85), with adjustments only in line spectra thresholds. Raw data processing was performed using DataAnalysis 5.2 (Bruker) with recalibration using sodium formate. RecalculateLinespectra with two thresholds (5,000 and 10,000) and subsequent FindMolecularFeatures (0.5 to 25 min, S/*N* = 0, minimal compound length = 8 spectra, smoothing width = 2, correlation coefficient threshold = 0.7) was performed. Bucketing was performed using ProfileAnalysis 2.3 (Bruker; 30 to 1,080 s, 100 to 6,000 *m*/*z*, Advanced Bucketing with 24 s, 5 ppm, no transformation, Bucketing basis = H^+^).

#### Data management and analysis: metabolic profiling of OSMAC cultivations

The complete data set was copied and processed using both line-spectrum thresholds (5,000 and 10,000). Features were deemed identical (and therefore sorted into the same bucket) if the retention time and mass precision were inside of 24 s and 5 ppm, respectively. Bucketing was performed on both sets simultaneously, resulting in a single table containing all buckets across all samples (under both thresholds). This table was subsequently curated: buckets were deemed present only if detected in the 10,000 samples (4,406 buckets did not meet this criterion and were deleted). To avoid “uniqueness” caused by compounds just above the detection threshold, entries from the 5,000 set were used for these buckets. The 10,000 entries were deleted. Thirty-seven buckets were not filled in this table and were subsequently deleted, resulting in a final table of 219 samples with 3,092 buckets. For barcoding, the conditions of each strain–medium combination were combined. Each feature was deemed present when it occurred in two (gray) or more (black) extracts of the strain–medium combination. Features that occurred only once were ignored as noise. Medium control extracts were produced twice for each chemical composition (exception for two media, where *n* = 1). For each chemical composition, a feature was deemed present when it occurred once (gray) or twice (black). The final table contained 2,747 buckets, which were used for barcode generation. Jaccard dissimilarities were computed (skbio.diversity.beta_diversity, metric = "jaccard") from the bucket table, and PCoA (skbio.stats.ordination.pcoa) was performed using scikit-bio (v0.7.0). PERMANOVA (skbio.stats.distance.permanova) was used to assess group differences with 999 permutations.

#### Data management: Trp induction

To minimize the inclusion of low-intensity signals close to the detection limit, the complete data set was copied and processed using both line-spectrum thresholds (5,000 and 10,000). Bucketing was performed on both sets simultaneously, resulting in a single table containing all buckets deemed identical across all samples (under both thresholds). This table was subsequently curated: buckets were deemed present only if detected in the 10,000 samples (3,673 buckets did not meet this criterion and were deleted). To avoid “uniqueness” caused by compounds just above the detection threshold, entries from the 5,000 set were used for these buckets; the 10,000 entries were deleted. Fifty-four buckets were not filled in this table and were subsequently deleted, resulting in a final table of 48 samples with 2,759 buckets.

For barcode preparation, a logical analysis was performed. Buckets filled in samples without tryptophan were colored gray if present. For samples spiked with tryptophan, the sample was compared to its non-spiked counterpart, and each bucket was categorized into Extinguished, Strong increase, Strong reduction, Light reduction, Present, Light increase, and Strong increase (SI–1.3). To achieve a readable representation, the buckets in the input table were sorted “decreasing” according to the presence/absence for each sample, then according to “sum present,” and subsequently for “present in media.” For PCA, we log2-transformed peak area intensities from the bucket table using scikit-learn (v1.3) in Python. Before PCA, undetected features were defined as zero, and samples were normalized to the injection volume wherever indicated. All visualizations were generated using Matplotlib.

### Compound isolation

The crude extract was dissolved in 10% MeOH to perform SPE, utilizing a 2 L column packed with Amberlite XAD-16 N and step gradient elution with 10%, 20%, 40%, 60%, 80%, and 100% MeOH. Based on LC-MS analysis, several fractions containing the compounds of interest were combined and evaporated to dryness. During isolation, semi-preparative and preparative HPLC were performed on Agilent 1100 HPLC systems equipped with external Gilson fraction collectors using H_2_O + 0.1% FA and ACN + 0.1% FA as mobile phases. Final purification was achieved by UPLC fractionation using the chromatographic method described in the UPLC-MS section, with fraction collection of 16 seconds per fraction, starting directly after injection.

### Structure elucidation by NMR

NMR spectra were recorded on a Bruker AVANCE III HD 700 MHz spectrometer operating at a ^1^H frequency of 700 MHz and a ^13^C-carbon frequency of 140 MHz. The instrument was equipped with a 5 mm TCI cryo probe head. COSY, TOCSY, ROESY, NOESY, HSQC, and HMBC experiments were recorded using standard pulse programs. All isolated samples were measured in DMSO-d6. Chemical shifts were referenced to the solvent signals (^1^H: 2.50 ppm, ^13^C: 39.5 ppm)

## Data Availability

The LC-MS/MS data sets generated during this study have been deposited in the MassIVE repository under accession MSV000102663 (DOI: 10.25345/C52R3PB4J). The NMR data sets of the isolated compounds have been deposited in the Natural Products Magnetic Resonance Database (NP-MRD) under accession numbers NP0354295 (pityriacitrin B) and NP0354296 (pityriacitrin B methyl ester). The metabolomics project, including processed data and metadata, is additionally available through the Fordatis repository (DOI: http://dx.doi.org/10.24406/fordatis/453). The custom scripts used for the plant growth-promoting trait (PGPT) analysis are publicly available at https://github.com/CeMaZu/Acidobacteriota-PGPT-analysis. Genomes were uploaded to NCBI and are deposited under the BioProject Number PRJNA1480456 under the following BioSample numbers: SAMN61030131 (FHG110202), SAMN61030132 (FHG110214), SAMN61030133 (FHG110511), and SAMN61030134 (*A. borealis* DSM 23886). All other data supporting the findings of this study are included within the article and its Supplemental Material.

## SUPPLEMENTAL MATERIAL

Supplemental material msystems.00828-26-s0001.pdf Supplemental text, tables, and figures.

Table S1 msystems.00828-26-s0002.xlsx Metadata for the 618 Acidobacteriota genomes included in the comparative genomic analysis.

ASM does not own the copyrights to Supplemental Material that may be linked to, or accessed through, an article. The authors have granted ASM a non-exclusive, world-wide license to publish the Supplemental Material files. Please contact the corresponding author directly for reuse.
